# Origin, Localization, and Immunoregulatory Properties of Pulmonary Phagocytes in Allergic Asthma

**DOI:** 10.3389/fimmu.2016.00107

**Published:** 2016-03-23

**Authors:** Franziska Hoffmann, Fanny Ender, Inken Schmudde, Ian P. Lewkowich, Jörg Köhl, Peter König, Yves Laumonnier

**Affiliations:** ^1^Institute for Anatomy, University of Lübeck, Lübeck, Germany; ^2^Institute for Systemic Inflammation Research, University of Lübeck, Lübeck, Germany; ^3^Division of Immunobiology, Cincinnati Children’s Hospital Medical Center, College of Medicine, University of Cincinnati, Cincinnati, OH, USA; ^4^Airway Research Center North (ARCN), German Center for Lung Research (DZL), Giessen, Germany

**Keywords:** macrophages, dendritic cells, monocytes, allergy and immunology, asthma, localization, functions

## Abstract

Allergic asthma is a chronic inflammatory disease of the airways that is driven by maladaptive T helper 2 (Th2) and Th17 immune responses against harmless, airborne substances. Pulmonary phagocytes represent the first line of defense in the lung where they constantly sense the local environment for potential threats. They comprise two distinct cell types, i.e., macrophages and dendritic cells (DC) that differ in their origins and functions. Alveolar macrophages quickly take up most of the inhaled allergens, yet do not deliver their cargo to naive T cells sampling in draining lymph nodes. In contrast, pulmonary DCs instruct CD4^+^ T cells develop into Th2 and Th17 effectors, initiating the maladaptive immune responses toward harmless environmental substances observed in allergic individuals. Unraveling the mechanisms underlying this mistaken identity of harmless, airborne substances by innate immune cells is one of the great challenges in asthma research. The identification of different pulmonary DC subsets, their role in antigen uptake, migration to the draining lymph nodes, and their potential to instruct distinct T cell responses has set the stage to unravel this mystery. However, at this point, a detailed understanding of the spatiotemporal resolution of DC subset localization, allergen uptake, processing, autocrine and paracrine cellular crosstalk, and the humoral factors that define the activation status of DCs is still lacking. In addition to DCs, at least two distinct macrophage populations have been identified in the lung that are either located in the airway/alveolar lumen or in the interstitium. Recent data suggest that such populations can exert either pro- or anti-inflammatory functions. Similar to the DC subsets, detailed insights into the individual roles of alveolar and interstitial macrophages during the different phases of asthma development are still missing. Here, we will provide an update on the current understanding of the origin, localization, and function of the diverse pulmonary antigen-presenting cell subsets, in particular with regard to the development and regulation of allergic asthma. While most data are from mouse models of experimental asthma, we have also included available human data to judge the translational value of the findings obtained in experimental asthma models.

## Current Understanding of Allergic Sensitization

Allergic asthma is a chronic inflammatory disease of the airways with high associated morbidity that is increasing in prevalence in western nations (1, 2). Patients suffer from airway hyperreactivity (AHR) and mucus overproduction resulting in recurrent episodes of chest tightness, breathlessness, wheezing, and coughing. In human, allergic asthma is also characterized by the recruitment of eosinophils, lymphocytes, and mast cells (2). Currently available therapeutics target the effector phase of the disease. They either reduce the inflammatory processes that drive the clinical symptoms or decrease the resistance in the airways and increase the airflow in the lung. In order to develop strategies that prevent allergic asthma development, we need a better understanding of the mechanisms underlying disease development and exacerbation. Most of our mechanistic insights into the pathways underlying the development of maladaptive immunity are derived from mouse models of experimental allergic asthma with all of their limitations (3). It is now generally accepted that the disease develops as an inappropriate Th2/Th17-adaptive immune response toward harmless environmental substances in genetically susceptible individuals (4). During sensitization, allergens enter the lung and reach the airway epithelium. The epithelial cell (EC) layer is not only a physical barrier but is activated by allergens *via* their protease activity (5–7) and through pattern-recognition receptors, in particular Toll-like receptors (TLR) 4 (8, 9). Allergen contact and loss of physical barrier integrity trigger the release of alarmins, including interleukin (IL)-33, high-mobility group box 1, uric acid, and adenosine triphosphate; cytokines, such as IL-1α, IL-25, thymic stromal lymphopoietin (TSLP), granulocyte–macrophage colony-stimulating factor (GM-CSF, CSF-2); and chemokines (e.g., CCL2), from the airway epithelium (6). These soluble mediators, in turn, recruit and activate cells of the innate immune system, such as macrophages, type 2 innate lymphoid cells (ILC2), and the pulmonary dendritic cell (DC) network (10). Among these, DCs are specialized in antigen uptake, processing, and presentation to naive T cells (11) and help them to differentiate into effector T cells, thereby bridging innate and adaptive immunity. In established allergic airway disease, pulmonary DCs are an important source of the chemokines CCL17 and CCL22, which attract effector T cells to the site of inflammation (12). In the sensitized lung, the release of IL-4, IL-5, and IL-13 from Th2 cells mainly contributes to the development of chronic inflammation, mucus overproduction, and AHR (13). In recent years, different pulmonary DC subsets in the lung have been identified. These subsets have unique localizations and functions indicating a division of labor regarding antigen uptake, activation of different T cell subsets, and activation of inflammatory innate effector cells. DCs are necessary and sufficient to induce adaptive immunity (14). However, recent reports show that alveolar macrophages (AM) and interstitial macrophages (IMs), the predominant phagocyte populations in the lung, play more important roles than previously recognized.

Thus, the entire phagocyte compartment with its complexity regarding developmental origin, tissue localization, and functional diversity has to be taken into account to gain a holistic view of the processes that drive the development of maladaptive immunity in allergic asthma.

The goal of this review is to detail recent advances in our understanding of pulmonary phagocytic cell subset biology regarding their origin, localization, and their functions in the context of allergic asthma.

## Diversity of Lung Phagocytes

While it is appreciated that in both humans and mice, pulmonary DCs and macrophages are the major phagocyte population that can function as professional antigen-presenting cells (APCs), most of the studies focusing on the composition of lung phagocytes have been performed in mice. Pulmonary APCs were originally described as a homogenous population of cells (15–18). It is now well appreciated that the mouse lung contains at least four different DC subsets and two macrophage subpopulations that can be distinguished by the expression of distinct surface markers, as well as monocytes (Table 1). DCs can be separated into CD11b^+^ and CD103^+^/Langerin^+^ conventional DCs (cDCs) (19, 20), plasmacytoid DCs (pDCs), and under inflammatory conditions, monocyte-derived DCs (moDCs). Lung macrophages can be divided into AMs and IMs. AMs comprise at least two distinct subsets, i.e., airway macrophages and macrophages truly residing in the alveolar space. In the rat, the available data suggest that they are of identical origin and that airway macrophages represent aged alveolar macrophages (AMs) with minor phenotypical and functional differences (21). Most studies refer to them collectively as AMs (22). In the alveolus, AMs are located in the alveolar lumen, while IMs are situated inside the lung interstitium. However, conflicting data concerning the expression of CD11b at the surface of IMs have been reported and suggest that IMs could be divided into two subpopulations (Table 1). Using a combination of conditional cell targeting and adoptive cell transfer, one study showed that blood monocytes transform into IMs and then migrate into the alveolar space, suggesting that IMs serve as an intermediate between monocytes and AMs (23). In addition to IMs, monocytes serve as precursors of monocyte-derived DCs (moDCs). Two types of circulating monocytes have been described, the classical Ly6C^hi^ and the non-classical Ly6C^lo^ monocytes (24, 25).

**Table 1:** **Phenotypic markers of murine pulmonary dendritic cells (DCs), alveolar macrophages (AMs), and interstitial macrophages (IMs)**.

	CD11b^+^ cDCs	CD103^+^ cDCs	pDCs	moDCs	AMs	IMs
**CD11b**	+++ (26)	+ (19, 20, 26)	+ (27)	+++ (26)	− (27, 28)	− (29)
						++ (30)
						+++ (31, 32)
**CD11c**	+++ (26)	+++ (26)	++ (26)	+++ (26)	+++ (26, 31, 33)	− (31, 32)
						+ (28, 30)
**CD64**	− (28)	− (26)	− (26)	+++ (26)	− (26)	+ (28)
					+ (34)	
**CD103**	− (19, 20)	+++ (19, 20)	− (20)	− (26)	+ (27)	?
**CD207 (Langerin)**	− (19, 20)	+++ (19, 20)	− (19, 20)	− (19, 20)	− (19, 20)	?
**CD317 (PDCA-1)**	− (27)	− (27)	+++ (27, 35)	− (27)	− (27)	?
**CXCR1**	− (19)	+++ (19)	?	?	?	?
**DNGR-1**	++ (36)	+++ (36)	− (36)	− (36)	− (36)	?
**F4/80**	++ (19, 37)	− (19)	− (28)	?	− (27, 38)	+ (28, 30)
					+ (28)	
**FcεR1**	− (26)	− (26)	− (26)	+++ (26)	− (26)	?
**Ly6C**	− (27)	− (27)	+ (26, 39)	+ (26)	+ (27)	?
	+ (28)					
**MHC-II**	+++ (26, 28)	+++ (26, 28)	+ (28)	+++ (26)	− (27)	++ (28)
					+ (26)	
**SiglecF**	− (26)	− (26)	− (26)	− (26)	+++ (26)	− (28)
**SiglecH**	− (27)	− (27)	+ (27, 39)	− (27)	− (27)	?
**Zbtb46**	+++ (40)	+++ (40)	− (40)	− (40)	− (40)	?

*+++, high expression level; ++, intermediate expression level; +, low expression level; −, not expressed; ?, unknown*.

**Figure 1:**
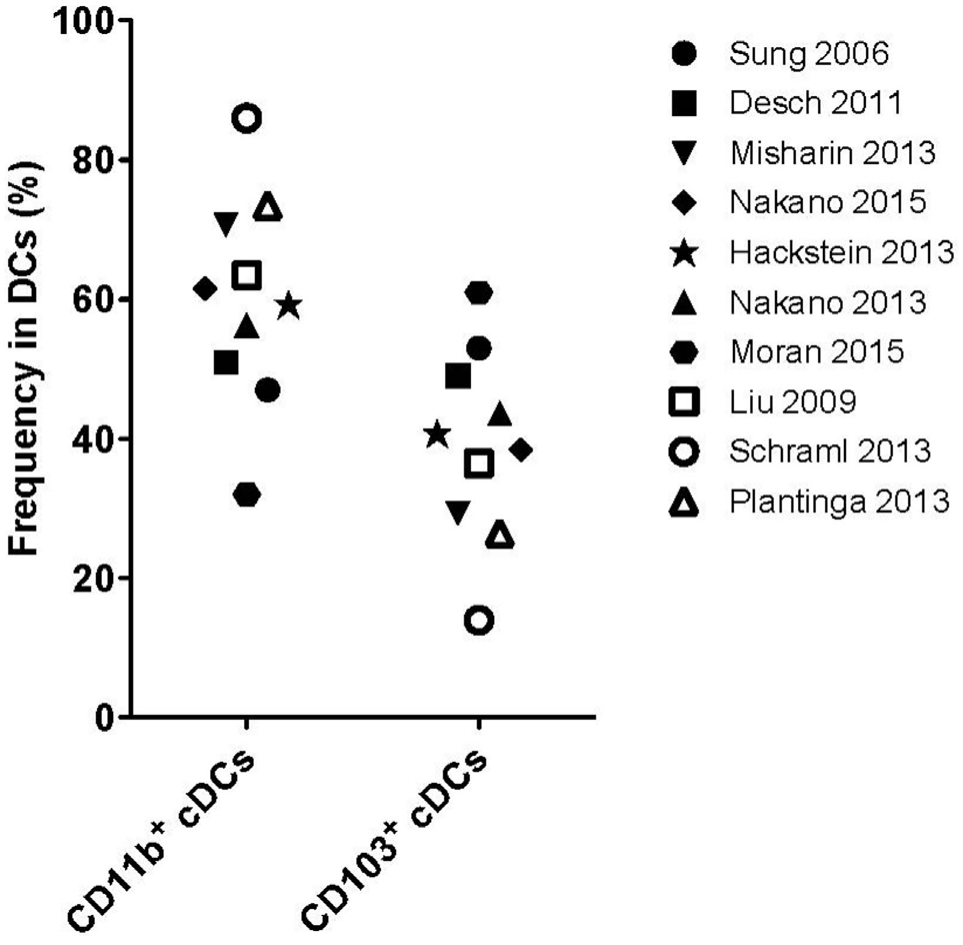
**Frequency of pulmonary conventional DC subsets**. Frequencies of CD11b^+^ or CD103^+^ cDC subsets as described in the studies outlined on the right. Open symbols: lung tissue digestion using collagenase D and closed symbols: lung tissue digestion using other collagenases.

The identification of lung DCs is complex and many markers are required to distinguish them properly (see Table 1). In most reports, CD11c, CD11b, MHC-II, SiglecF, CD64, and CD103 have been used as DC subset-defining markers on lineage negative cells. Additional markers were reported in individual studies (26, 28, 36, 41, 42), pointing toward the heterogeneity of flow cytometry-based gating strategies to distinguish DC subsets. As a result, absolute numbers of a given DC subset in the lung, and even some functional properties, markedly vary between studies. Further complexity arises from the use of different cell isolation procedures, which may liberate DCs from different tissues with different efficiencies. Indeed, a recent study has shown that cell isolation followed by flow cytometric analysis results in up to 70-fold underestimation of resident memory CD8^+^ T cells in different organs (including the lung), when compared to quantitative immunofluorescence microscopy (43). In contrast, the cell numbers in spleen and lymph nodes were essentially the same for both techniques (43). Given these challenges, it is perhaps not surprising that while most studies report cDCs as the dominant DC population in the lung, varying numbers (20, 28) and ratios of CD11b^+^ cDCs to CD103^+^ cDCs (from 1:1 up to 4:1) (20, 26, 28, 36, 41, 42, 44–47) have been reported (Figure 1).

In humans, Demedts et al. described three pulmonary DCs subset, i.e., two “myeloid” DC (mDC) populations and one population of pDCs. The mDC1 subset expresses BDCA1 (CD1c), whereas the mDC2 subset is BDCA3^+^ (CD141^+^). The pDC express BDCA2 (CD123) (48). More recently, a detailed characterization of the DC subsets has been described in human blood and skin [reviewed by Reynolds and Haniffa (49)]. These studies confirmed the existence of two subsets of cDCs, BDCA1/CD1c^+^ cells (termed cDC1s) and BDCA3/CD141^+^ cells (termed cDC2) (49). Transcriptome profiling and functional studies showed that the cDC1 population is equivalent to the murine CD103^+^ cDC subset (50, 51). Similar studies revealed that cDC2 are similar to the murine CD11b^+^ cDC population with whom they share additional markers such as CD11b, CX3CR1, and SIRP-a (52). Interestingly, transcriptome mapping analysis of human and mouse non-lymphoid tissue suggests that the mouse pulmonary CD11b^+^ cDCs are heterogeneous and comprise cells that are related to both human CD14^+^ DCs and BDCA1/CD1c^+^ cDC2 (50). In agreement with this profile, the mDC1 population has originally been described as a mixed CD14^−^/CD14^lo^ population of DCs (48).

These results demonstrate the inherent variability that can be introduced by different isolation/analysis techniques and highlight the fact that differences observed between studies and groups may be more artifactual than accurate representations of true biological differences. As such, caution should be applied when comparing results from different studies.

### Origin of Lung DCs

Surface markers do not unambiguously distinguish DC subpopulations. Some populations, i.e., CD11b^+^ cDCs and moDCs, have largely overlapping marker profiles, despite the fact that these DC subsets have different functions and derive from different precursors. An alternative approach to identify different DC populations is based on their cellular origin. In the next section, we will provide an update on our current understanding of the origin of lung phagocytes and recent approaches developed for lineage tracking.

#### Generation of Pulmonary CD103^+^ and CD11b^+^ cDCs and pDCs

**Figure 2:**
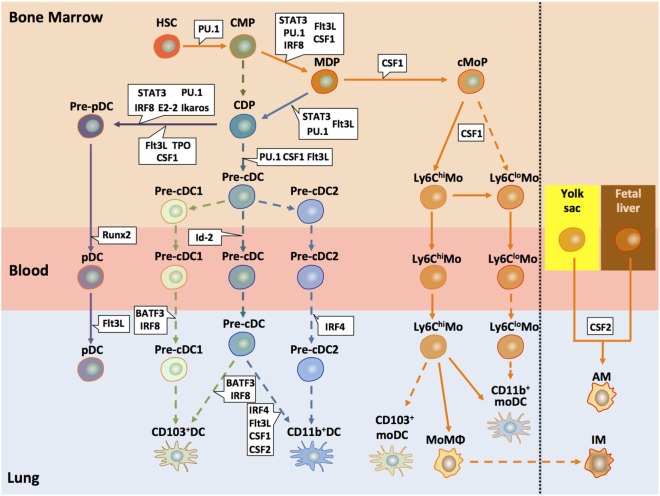
**Development of pulmonary DC and macrophage subsets**. This model of pulmonary DC and macrophage subset differentiation in mice summarizes recent findings suggesting early lineage commitment of cDCs in the BM and differentiation of monocytes into different population with DC, macrophage, or suppressive functions. All DC subsets present in the lung originate from hematopoietic progenitors (HSC) that differentiate into a common myeloid progenitor (CMP). Such CMPs further differentiate to a common DC progenitors (CDPs) or macrophage DC progenitors (MDPs) (53, 62, 65). MDPs give rise to a common monocyte precursor (cMoP). In a CSF-1-dependent mechanism, Ly6C^hi^ monocytes develop, which can further differentiate into Ly6C^lo^ monocytes. Such Ly6C^lo^ monocytes may also derive directly from cMoPs. Both monocyte populations can enter the lung and become monocyte-derived DCs, macrophages, or suppressor cells (25, 62). CDPs also serve as precursors for pDCs and pre-cDCs. Recent studies suggest that the two cDC populations deriving from the pre-cDC progenitor, i.e., CD103^+^ cDCs and CD11b^+^ cDCs, arise already in the bone marrow as pre-cDC1/cDC2 subtypes (66). One study suggested that pulmonary monocytes may differentiate into pulmonary CD103^+^ and CD11b^+^ DC; however, it is unclear whether such cells are phenotypically and functionally identical to CD103^+^ and CD11b^+^ cDCs (67). Activation of defined transcription factors (in blue) at distinct time points is critical for lineage commitment of the different DC precursors (68). During the early developmental stages, important transcription factors include STAT3, IRF8, and PU.1. At later stages, E2-2 is decisive for pDC commitment of CDPs. BATF3 and IRF8 are associated with the CD103^+^ cDC and IRF4 with the CD11b^+^ differentiation. In addition to the transcription factors, several growth factors (in green) play key functions in the development of pre-cDCs and the different DC subsets, in particular Flt3L, CSF-1 (M-CSF), and CSF-2 (GM-CSF). The lung contains two major macrophage populations, i.e., alveolar and interstitial macrophages (AMs and IMs, respectively). It is now well appreciated that AMs derive from yolk sac and fetal liver progenitors that colonize the embryonic lung and are maintained by self-renewal at steady state (62). The origin of IMs remains elusive. Some data suggest that they represent monocyte-derived macrophages (69, 70). Solid arrows depict pathways that are found by independent studies; dashed arrows show pathways that are rely on a single study or that are controversial. HSC, hematopoietic stem cells; CMP, common myeloid progenitor; MDP, monocyte–macrophage DC progenitor; CDP, common DC progenitor; cMoP, common monocyte progenitor; Mo, monocyte; MoMΦ, monocyte-derived macrophage; cDC, conventional DC; moDC, monocyte-derived DC; AM, alveolar macrophage; IM, interstitial macrophage.

Dendritic cells in the lung originate from hematopoietic stem cells (HSCs) in the bone marrow (BM) that give rise to a macrophage DC progenitor (MDP) (53). This MDP then differentiates either into the common monocyte progenitor (cMoP) (54) or the common DC progenitor (CDP) (45, 55). However, at this point, it is not entirely clear whether CDPs may also develop without the intermediate MDP step (56). CDPs give rise to pre-DCs that can differentiate into cDCs and pDCs but lose their capacity to differentiate into monocytes or macrophages (57, 58). In the classical model, developed using mouse data, CD11c^+^ MHC-II^−^ CD135^+^ pre-DCs (19, 59) differentiate into lung tissue-resident CD103^+^ and CD11b^+^ cDC subsets depending on different transcription and growth factors (see below) (53, 60–62). More recently, two alternative models have evolved based on single-cell mRNA sequencing (63) or chromatin profiling (64) of mouse cells. They suggest that the commitment into the different cDC subsets can already occur at the pre-cDC stage, since they found precommitted cells in the pre-DC pool of the BM (63) (Figure 2). Such pre-cDCs can be locked into their terminal differentiation state (CD103^+^ and CD11b^+^ cDCs) through an IRF8/BATF3-dependent amplification loop (64). Although pre-DCs can enter the lung and cells phenotypically resembling such pre-DCs have been found in the lung, the differentiation into the two cDC populations has not yet been shown *in situ* (19, 62).

The origin of human DCs is still incompletely understood. Originally, most studies focused on *in vitro* development of progenitor cells in the presence of CSF-2 (GM-CSF) and/or IL-4, but such approaches are of limited relevance as they reflect the lineage development of moDCs but not of steady-state cDC subsets (71). More recent studies have shown that DCs arise from BM progenitor cells, in particular granulocyte/macrophage progenitors and multi-lymphoid progenitors, but a complete picture, including the identity of intermediate cells, is missing (71). However, the nature of the cytokines (CSF-2 and Flt3L) and of the transcription factors (e.g., Ikaros, PU.1, IRF4, IRF8, and BATF3) involved in human DCs development [reviewed in Ref. (71)] suggests that the mechanisms described for the development of mouse cDCs could be similar in humans.

#### Cytokines That Control cDC Differentiation

The development of DC precursors and differentiated DC subsets is dependent on several signaling molecules. In the BM and in peripheral organs, Fms-like tyrosine kinase receptor 3-ligand (Flt3L) drives the development from early progenitors into DC precursors (pre-DCs) and DCs (19, 45, 72–74). Importantly, Flt3L is required for the development of cDCs and pDCs but not for moDCs (75). Furthermore, pre-DCs are present at lower numbers in Flt3L^−/−^ than in wild-type (WT) mice (19), suggesting that Flt3L drives the proliferation and differentiation of pre-DCs *in situ* (62). However, the cDC subsets are not equally dependent on Flt3L for their differentiation since ~10% of CD11b^+^ cDCs remain in the lungs of Flt3L^−/−^ animals, whereas CD103^+^ cDCs are almost completely absent (19, 26). In line with these observations, lung CD103^+^ cDCs depend exclusively on Flt3L and its downstream target the Phosphatidylinositol-3-kinase gamma (PI3Kγ) for their development. Pulmonary CD11b^+^ cDCs and cDCs in other tissues depend on both Flt3L and other yet unknown cell signals for their terminal differentiation (76).

CSF-2 is another important factor for the differentiation of DCs in non-lymphoid tissues, which acts synergistically with Flt3L (73). CSF-2 exerts its function through CSF-2 receptor that is expressed on MDPs and CDPs. The exact contribution of CSF-2 for pulmonary cDC differentiation is not entirely clear. In one study, CSF-2 has been described as a critical factor for the homeostasis of cDCs in the lung (77). The authors found lower numbers of resident cDCs in lung tissue as well as migratory CD103^+^ cDCs in the lung draining lymph nodes in Csf2^−/−^ mice (77). In contrast, others showed that CSF-2 did not affect pulmonary cDC numbers but was critical for the surface expression of CD103 (78, 79) and cross-presentation (80).

In addition to Flt3L and CSF-2, several other cytokines contribute to cDC differentiation [for detailed review, see Ref. (65)]. CSF-1 (M-CSF) is primarily known for its function as a regulator of macrophage survival, proliferation, and differentiation (81) With regard to cDC differentiation, CSF-1 acts on CDPs and may strengthen the Flt3L signal. Since CSF-1 receptor (CD115) expression is lost upon differentiation from pre-cDCs into CD103^+^ cDCs, but not into CD11b^+^ cDCs, CSF-1 partly regulates their differentiation and survival (19). Lymphotoxin-β plays an important role in the differentiation of lymphoid cDCs; its role for the differentiation for cDCs in non-lymphoid organs remains elusive (65). Finally, TGF-β has been described as an important cytokine for Langerhans cell differentiation (65). Its role for pulmonary cDCs differentiation has yet to be explored.

#### Transcriptional Regulators of cDC Development

In addition to cytokines, several transcription factors regulate the development of cDCs. The development of CD103^+^ cDC critically depends on the transcription factors BATF3 (82, 83), IRF8, and Id2 (Figure 2) (19). For the homogenous population of lung CD103^+^ cDCs, it was reported that their development is similar to CD8α^+^ DCs in lymphoid organs (19, 42). The situation for CD11b^+^ cDCs is less clear. Their development is critically dependent on the transcription factor IRF4 (52). However, many studies have not taken into account that even at steady state, CD11b^+^, CD11c^+^, and MHC-II^+^ cells in the lung are a mixed population consisting of CD11b^+^ cDCs, short-lived Ly6C^hi^, and long-lived Ly6C^lo^ CD11b^+^ moDCs. At this point, it remains unclear whether peripheral and CD11b^+^ cDCs and their counterparts in lymphoid organs have the same origin (47, 74, 84–86).

### Lineage Tracking of Pulmonary APCs

#### Conventional DCs

As mentioned above, the distinction of DC subsets by surface marker expression is difficult. Therefore, new tools have been developed to allow lineage tracing of different populations to clearly assign DCs to a single subset. Recently, the zinc finger transcription factor Zbtb46 has been identified and shown to be selectively expressed by all cDCs and pre-DCs but not in pDCs and macrophages (85). However, this marker is downregulated after DC stimulation and is also expressed on endothelial cells, early erythroid progenitors, and IL-4-stimulated monocytes (40, 85, 87). Another study (36) described DNGR-1 (*Clec9A*) as a bona fide marker of mouse cDC precursors. The authors found high levels of DNGR-1 expression in CD8α^+^ and CD103^+^ CD11b^−^ cDCs and lower levels in pDCs (88–90). Further, Schraml et al. (36) presented a model to identify CDP, pre-cDC, and their progenies using the Clec9a^+/cre^Rosa^+/EYFP^ mouse. This mouse allows, by recombination during cell development, the labeling of DNGR-1^+^ cells and their progeny by the enhanced yellow fluorescent protein (eYFP). In the lung, >95% of the eYFP^+^ cells were cDCs or pDCs with low numbers of eYFP positive moDCs. In agreement with their high expression of DNGR-1 (41, 89), CD103^+^ cDCs were homogenously positive for the lineage marker, confirming that they derived from CDP progenitors and pre-DCs. However, not all cells within the DC lineage expressed eYFP despite DNGR-1 expression in precursors, due to a general problem within the cre/loxP system during DC development. Therefore, the incomplete eYFP expression observed in the CD11b^+^ DC population points toward a mixed population comprising CD11b^+^ cDCs and cells from another lineage resembling CD11b^+^ cDCs but does not formally prove this hypothesis.

Regarding CD11b^+^ DCs, it was recently reported that the complement C5aR1/CD88 is highly expressed on moDCs but at lower levels on CD11b^+^ cDCs (42, 91). In contrast, only the CD103^+^ and CD11b^+^ cDC populations express the dipeptidyl peptidase-4 (DPP4)/CD26 (42) allowing the authors to distinguish cDCs (CD26^+^ C5aR1^−^) from moDCs (CD26^−^ C5aR1^+^). However, the use of CD26 in combination with C5aR1 seems to be restricted to the C57Bl/6 background and does not apply to Balb/c mice (42).

#### Plasmacytoid DCs

Plasmacytoid DCs account for a minor population under steady-state conditions and can be defined by the expression of intermediate levels of CD11c and MHC-II, BM stromal antigen 2 (mPDCA-1; CD317), SiglecH, B220, and Ly6C, which is also part of the Gr-1 marker (20, 92–94). In contrast to cDCs, pDCs differentiate from the CDP into a mature pDC (Figure 2) in the BM before they migrate to other organs (95). As for cDCs, Flt3L is also important for the pDC development through the induction of STAT3 and the induction of various transcription factors among which E2-2 is considered to be highly specific for the pDC lineage (96, 97). It has been shown that cell signals induced by CSF-2 in CDP counteracted the Flt3/STAT3 pathways in a STAT5-dependent manner (96). In addition, other cytokines, such as CSF-1 (98), and thrombopoietin (57) synergize with Flt3L during pDC differentiation. Furthermore, IL-7 can complement Flt3L signaling (99).

#### Monocytes

Like cDCs, monocytes start their development in the BM from HSCs that generate MDP, which give rise to cMoPs (54). Out of such cMoPs, the Ly6C^hi^ monocyte population is generated that can convert into Ly6C^low^ monocytes (Figure 2). The classical Ly6C^hi^ CC-chemokine receptor 2 (CCR2)^hi^ (Gr-1^hi^) monocytes emigrate to the sites of ongoing inflammation, while the non-classical Ly6C^lo^ CCR2^lo^ (Gr-1^lo^) monocytes express high amounts of CX3CR1 and patrol the vascular wall (100–102). The classical Ly6C^hi^ monocytes have a very short half-life of about 1 day (45, 103). As outlined above, they can serve as precursors for the Ly6C^lo^ population (86, 103–105), but this is still controversial (25). Further, Ly6C^hi^ monocytes can differentiate into short-lived Ly6C^hi^ CD11b^+^ moDCs (29, 34, 106). In contrast, Ly6C^lo^ monocytes give rise to long-lived Ly6C^lo^ CD11b^+^ moDCs. However, at steady state, Ly6C^+^ monocytes that migrate into the lung not necessarily differentiate into DCs but can further migrate to the lymph nodes without differentiation (34), suggesting that additional stimuli must exist that drive monocyte differentiation in the lung tissue.

#### Monocyte-Derived DCs

At steady state, pulmonary moDC numbers are very low (26, 52). Environmental factors (e.g., cigarette smoke or ozone) or airborne allergens, including house dust mite (HDM), can trigger the production of cytokines and chemokines. HDM comprises bacterial and fungal contaminants that activate pattern-recognition receptors of the TLR and the C-type lectin families. Among the chemokines, CCL2 drives the migration of monocytes to the lung in a CCR2-dependent manner (59, 84, 107–109). These CCR2^+^ precursors can give rise to the moDC subset in the presence of both CCL2 (26) and CSF-1 (19) (Figure 2). In the lung, moDCs are characterized by the expression of CD11c, CD11b, Ly6C, CD64, and FcεR1 (26). However, mature moDCs tend to lose the Ly6C marker (26, 84). Of note, using an adoptive transfer model, the Randolph’s laboratory showed that monocytes can serve as precursor for pulmonary DCs. A pulmonary CD103^+^/Langerin^+^ subset can arise from a Ly6C^hi^ CCR2^hi^ monocyte precursor, while a CD11b^+^ moDC subset can arise from Ly6C^lo^ CCR2^lo^ monocytes in the lung (67).

#### Alveolar Macrophages

Alveolar macrophages derive from yolk sac macrophages or fetal liver monocytes. These cells enter the lung during embryonic development and colonize the alveoli in the first days after birth (38). While early monocyte precursors were recruited to the lung in animals lacking CSF-2, early AM commitment was absent in these animals. Moreover, short-term CSF-2 therapy restored AM development for weeks, although such AMs had a rather immature phenotype (38). These results suggest that local production of CSF-2 in the lung is required for both the transition from monocyte precursors and full maturation of AMs and that once AMs begin to populate the lung, they self-maintain throughout the lifetime of the host.

Under inflammatory conditions, blood monocytes are recruited into tissues and differentiate into macrophages (34, 110) as exemplified in LPS-induced lung inflammation (111). This macrophage population is expressing high levels of CD11b and low levels of CD11c, which is in contrast to resident CD11c^hi^CD11b^lo^ AMs, suggesting that monocyte-derived macrophages rather resemble CD11b^+^ IMs than AMs. This view is in accordance with the finding that monocyte-derived IMs serve as a transition state for AMs (23). However, in a model of HDM-driven allergic asthma, the increased number of AMs resulted rather from the proliferation of existing, tissue-resident AMs than from the differentiation of circulating monocytes (112). Together, the available data suggest that in addition to the proliferation of self-maintaining AMs, the alveolar compartment may also be filled up with macrophages of monocytic origin.

#### Interstitial Macrophages

Interstitial macrophages are a rather poorly defined pulmonary cell population that expresses the F4/80 and CD11b markers but lack the expression of CD11c (31, 32). In addition to this population, a similar cell type termed non-migratory myeloid cell, that is, CD11b^+^ Gr-1^int^ F4/80^+^ has been described (69, 70). Up to now, the origin of IMs is unclear. They may either arise from a common unknown precursor, or, more likely, derive from the macrophage or monocytic lineage (see above). In line with the view that IMs derive from the monocyte/macrophage lineage, both IMs and cells of the monocyte/macrophage lineage effectively suppress Th2 responses (31, 70).

Understanding the developmental origin of the different pulmonary DC subsets will help to reliably identify individual DCs subsets by cell-specific markers that may also prove useful as therapeutic targets allowing DC subset-specific manipulation. Moreover, such knowledge may provide preliminary insights into the functional differences of the distinct DC subsets and their roles in driving and maintaining the maladaptive immune response that are central to asthma pathogenesis.

## Localization of APC Subsets in the Lung

Pulmonary cDCs are critical for allergen uptake in the lung, its processing, presentation in the context of MHC-II, and the subsequent activation of naive CD4^+^ T cells in the draining lymph nodes. However, a detailed spatiotemporal resolution of initial allergen contact and capture is still lacking. A prerequisite for such studies is a detailed picture of the localization of the different DC subsets within the lung under steady-state conditions. The lung consists of different compartments, i.e., the airways, veins, arteries, the alveolar compartment, and the pleura. Interestingly, lymph vessels that are required for DC migration to the draining lymph node run with veins, around airways, and in the connective tissue between airways and pulmonary arteries (113), which make these compartments likely areas for DC location.

Early immunohistochemical studies describing DCs in the lung used MHC-II as the sole DC marker. Such studies reported DCs in many compartments of the lung, including the large conducting airways, lung parenchyma, alveolar compartment, pleura, perivascular space, and inside pulmonary lung vessels (17, 114). However, in addition to DCs, type II pneumocytes and IMs also express MHC-II, demonstrating that MHC-II alone is not sufficient as a DC marker and that further studies are needed to clarify the localization of DC subsets. Until now, no comprehensive study exists that describes the localization of all DC subsets in the lung. This section summarizes the available data regarding the localization of individual pulmonary DC subsets.

### CD103^+^ cDCs

MHC-II^+^ intraepithelial DCs (115, 116) described in the trachea of the rat were later equated with the CD103^+^ cDC subset in the lung of mice (108). Although immunohistochemical analysis of the CD103^+^ cDC subset showed its localization close to the airway epithelium, cell bodies were rarely observed in the epithelium itself (20). The apparent discrepancy between the intraepithelial network in the rat trachea that was described to sample antigen through the epithelial barrier (115, 116) and the localization of CD103^+^ DCs below the epithelium was later explained by the observation that CD103^+^ DCs extend their dendrites through the epithelial layer and into the airway lumen (20). This finding supported the observation that CD103 (alpha integrin) and beta7 integrin can interact with E-cadherin that is expressed at the basal side of ECs (20). Additionally, CD103^+^ cDCs have a higher expression of the tight junction proteins Claudin-1, Claudin-7, and Zonula Occludens (ZO)-2 as compared to other DC subsets. This might facilitate the extensions of dendrites through the epithelial barrier into the airway lumen (20).

Two-photon analyses of precision-cut lung slices from CD11c-eYFP mice showed approximately four times more subepithelial than intraepithelial DCs (117). Nevertheless, DC extensions to the airway lumen were exceptional observations in a few experiments. This finding was supported by a similar study using OVA-sensitized mice, where DCs extended no protrusions into the airway lumen, while a high activity of transepithelial antigen uptake in the alveolar region was observed (118). In addition, CD103^+^ cDCs can be found on the parenchymal side of arteriole walls underneath the vascular endothelial cells and potentially attached to the basal lamina, but they are absent in the alveolar region (20). No data are available for pulmonary veins.

### Monocytes and CD11b^+^ DC Populations (CD11b^+^ cDCs and moDCs)

Monocytes that reside in the lung at steady state can take up antigen and migrate to the draining lymph node (34). However, data regarding their localization in the lung are scarce. Most of the studies dealing with lung DCs focused on functional cell analyses but not on spatial distribution. The first study describing the localization of CD11b^+^ DCs used a flow cytometric method. The lung was divided into main conducting airways (trachea and main bronchi) and lung parenchyma (peripheral third of the lung) to determine the localization of different APC subsets (18). CD11b^+^ DCs were found in the lung parenchyma and in the main conducting airway fraction (18). However, this approach did not take into account that the distal part of the lung still contains airways and blood vessels. Upon infection with *Bacillus anthracis*, a CD11b^+^ DCs population was observed in the alveolar region (119). A study based on immunohistochemistry showed CD11b^+^ DCs in the perivascular regions but only few cells in the epithelial region (20). Another group showed that in PBS-treated mice, CD11b^+^ DCs can be found around the airways up to a distance of about 200 μm (118). Thus, the available data suggest that CD11b^+^ DCs can be widely distributed within the lung.

More recently, an elegant approach has been used that combines *Csfr1*-EGFP (120), *Cx3cr1*-EGFP (121), and *Csf1r*-ECFP^tg/+^ mice (MacBlue), the latter of which lacking a conserved distal element of the *Csfr1* promoter. Blood monocytes of the MacBlue mice strongly express ECFP, whereas most lung tissue macrophages are ECFP negative (122). The authors used such mice and monitored lung monocyte trafficking *in situ* in explanted lungs by two-photon imaging (123). They found that monocyte-derived cells are located at the interface between blood and airways, whereas lung DCs are strictly located in the airways.

### Plasmacytoid DCs

Similar to the moDC subset, very limited data are available for the localization of pDCs. Immunohistochemical staining suing the Gr-1 and B220 markers identified pDCs mainly in the alveolar interstitium (20, 92). However, pulmonary pDCs comprise a very small fraction in the naive lung (92), which makes them difficult to accurately identify under steady-state conditions.

### Alveolar Macrophages

In contrast to all other populations described so far, AMs are not located in the lung tissue but reside in the airways and the alveolar lumen (18, 21, 33, 124). Soroosh et al. (33) also reported tissue-resident AMs. They defined tissue-resident AMs as cells that are not removed by a bronchoalveolar lavage (BAL). Unfortunately, they did not perform any histological studies (33). Thus, it remains unclear whether such AMs were not washed out because they were located inside the lung parenchyma or due to their attachment to the alveolar wall. Interestingly, studies in iron oxide-treated mice or hamsters demonstrated that AMs adhere to the surface of airway ECs (125, 126). More recently, it was shown that AMs can be divided into alveolus-adherent and non-adherent populations (127), supporting the view that the reported tissue-resident macrophages are attached to the airway and alveolar walls.

### Interstitial Macrophages

As outlined above, IMs are poorly defined (31, 32). Only one paper examined the localization of IMs based on the presence of F4/80 and the absence of the CD11c markers. They found these cells exclusively in the alveolar interstitium close to DCs (31). It is likely that not all pulmonary IM populations were identified in this paper. Clearly, further studies are needed to get a better picture of the spatial distribution of all IMs in the lung.

Taken together, our picture of pulmonary DC subset and IM localization is rather sketchy and incomplete. Unraveling the exact localization of pulmonary phagocytes will provide preliminary insights, in which areas in the lung-specific DC subtypes take up antigen during initial allergen encounter. Further, it is a crucial step to identify proximity or even physical interactions between specific DC subtypes, other lung tissue-resident cells, and incoming inflammatory cells that act in concert to regulate DC function in response to allergen contact. Thus, combining knowledge of cell origin and localization will help us to understand the specialized functions of the different DC subsets. In the following section, we will summarize our current understanding of APC subset functions during the different phases of allergic asthma.

## Lung DC Subset Functions During Allergen Sensitization and the Effector Phase

**Figure 3:**
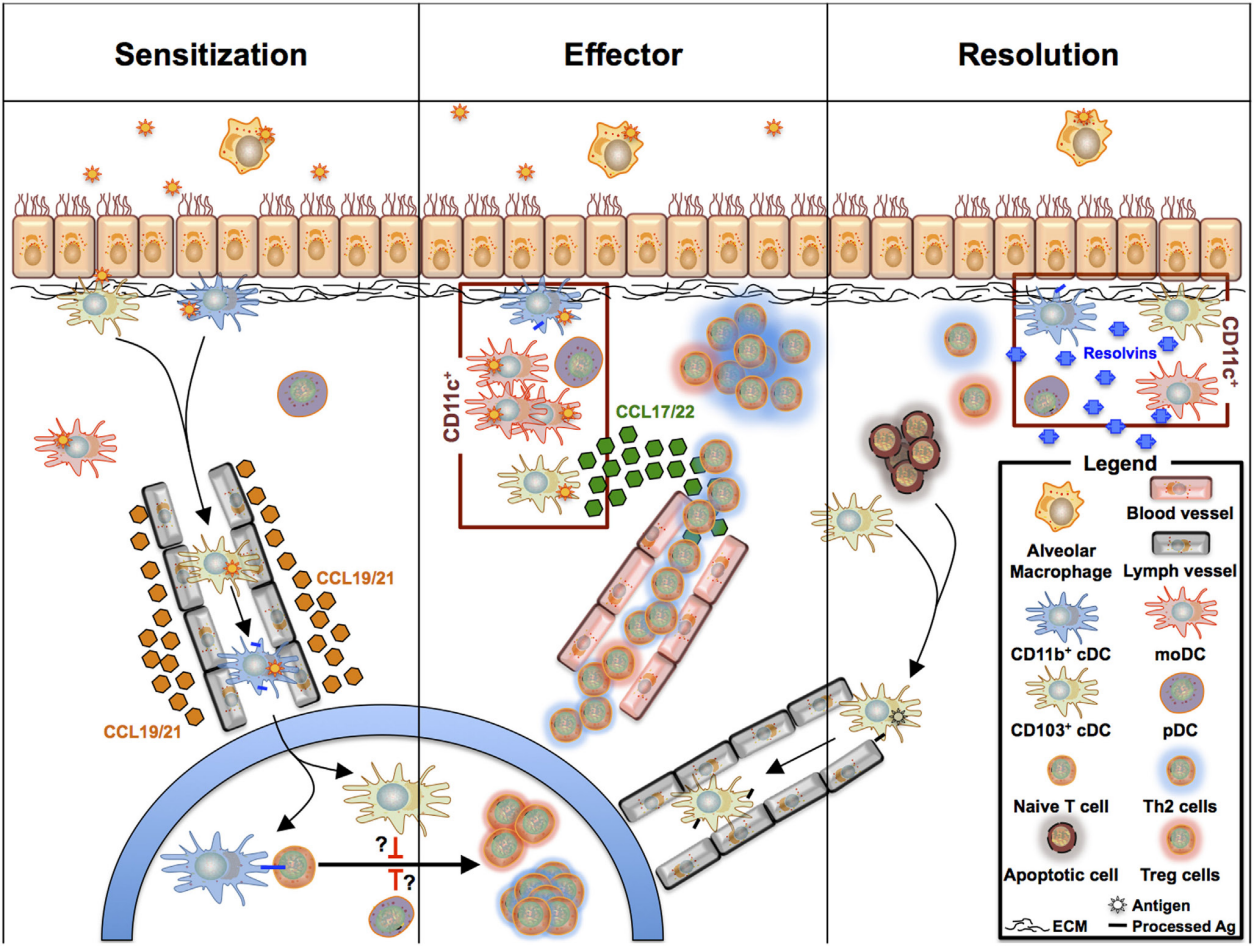
**Overview of pulmonary phagocyte functions during sensitization, effector phase, and resolution during allergic asthma-driven inflammation**. CD11b^+^ cDCs take up antigen and migrate to the draining lymph nodes, where they drive Th2 polarization. The nature of the accessory DC subsets regulating the CD11b^+^ cDC/T cell crosstalk is still unclear as pDCs and CD103^+^ cDCs can promote Treg cell polarization and tolerance. During the allergic effector phase, all CD11c^+^ DC subsets can activate effector T cells that have homed from the lymph nodes into the lung tissue. In this scenario, CD103^+^ cDCs may serve as the driving force recruiting the T cells to the tissue, whereas moDCs are orchestrating the inflammatory response supporting T cell activation. Following the effector phase, tissue inflammation will be resolved and lung remodeling pathways are activated. CD11c^+^ DCs also contribute to the resolution phase by releasing resolvins and other anti-inflammatory molecules. Further, they take up apoptotic cells.

Dendritic cells are well appreciated for their critical roles during allergen sensitization and the effector phase of allergic asthma. Numerous studies have summarized their multiple roles in allergen uptake, cytokine, chemokine production, and most importantly, their capability to induce differentiation of naive T cells toward the Th1, Th2, Th17, and Treg subtypes (7, 128). Pulmonary DCs were long considered as a homogeneous population that shares functional similarities with BM-derived DCs. However, it is now well appreciated that at least three different pulmonary DCs exist that originate from CDPs and cMoPs (see above) and fulfill different tasks in the development of allergic asthma and the regulation of allergic inflammation. Accordingly, new data regarding the function of each subset arise continuously (Figure 3).

### CD103^+^ cDCs

CD103^+^ cDCs have been shown to efficiently take up viral particles (129). Furthermore, CD103^+^ cDCs have been associated with the antiviral response of CD8^+^ T cells due to their ability to cross-present antigens (130) and their antigen MHC-I-loading machinery, which is superior to CD11b^+^ cDCs (129). Interestingly, the ability of CD103^+^ cDCs to migrate to the lymph node after influenza infection is dependent on the secretion of CSF-2 from ECs (131). However, CD103^+^ cDCs are less active in antigen uptake compared to other DC subsets in an HDM-dependent asthma model (26). CD103^+^ cDCs express the tight-junction proteins Claudin-1, Claudin-7, and ZO-2, which allow them to form tight junctions with airway ECs. These findings suggest that they sample the airway lumen without barrier damage (20). As indicated above, imaging data are lacking to support this notion. In the gut, macrophages were shown to sample the lumen and transfer allergen–MHC-II complexes to CD103^+^ cDCs in a connexin 43-dependent manner (132). Interestingly, a subset of AMs has recently been described that uses connexin 43 to build syncytial communication with ECs (127). However, transport of antigen from AMs to CD103^+^ cDCs has not been described at the mucosal interface of the lung. The role of CD103^+^ cDCs in Th2/Th17 skewing, which is characteristic for allergic asthma, is still controversial (26, 106). Nakano et al. showed that CD103^+^ cDCs can effectively uptake antigen, migrate toward draining lymph nodes, and drive Th2 responses (106). In contrast, Plantinga et al. could not confirm this finding (26). The nature of the allergen (OVA, cockroach, or HDM) and the amount used during the sensitization phase may explain the different findings. In contrast to the involvement of CD103^+^ cDCs in Th2 differentiation of T cells, other studies showed that CD103^+^ cDCs promote a Th1-biased response (133). Furthermore, CD103^+^ DCs were also suggested to play a prominent role in tolerance induction. It was shown that they induce *de novo* differentiation of Tregs through the production of retinoic acid and PPARγ (134, 135). This is in agreement with recent evidence showing that CD103^+^ cDCs can limit the inflammatory response during OVA- or HDM-driven asthma (136). Lastly, CD103^+^ cDCs are a major source of CCL17 and CCL22 (26, 137), suggesting a significant role in homing of activated T cells into the pulmonary compartment.

### CD11b^+^ cDCs

Together with CD103^+^ cDCs, CD11b^+^ cDCs are the major DC population in the lung. They are very efficient in allergen uptake *ex vivo* as well as *in vivo* (133) and have been identified as the major “migratory” DC subset, translocating quickly to the mediastinal lymph nodes after allergen exposure (26). Consequently, they are thought to be essential for the allergen-induced Th2 response (26, 133, 138). In addition to their role in driving Th2 differentiation, CD11b^+^ cDCs drive the induction of Th17 responses in a fungal infection model (52). In a HDM-mediated asthma model, CD11b^+^ cDCs promote Th2 and Th17 differentiation *via* a dectin-2-dependent mechanism (139).

Compromising the epithelial barrier integrity triggers the release of epithelial danger signals and cytokines, such as CSF-2, TSLP, IL-25, and IL33. CSF-2 has not only been shown to promote the survival and homeostasis of CD11b^+^ cDCs (77) and their recruitment to the lung (131) but also to license CD11b^+^ cDCs for Th2 differentiation in a *Blomia tropicalis* dust mite model (140). TSLP upregulates the expression of OX40L at the surface of CD11c^+^ cDCs (141), thereby enhancing DC-driven Th2 differentiation (142). Furthermore, neutralization of TSLP decreases the expression levels of CD40, CD80, and CD86 costimulatory molecules on the surface of CD11c^+^ DCs (143). Unfortunately, the authors did not define the exact subtype of DC that was affected by this treatment. More recently, OX40L expression has been described at the surface of CD11b^+^ but not of CD103^+^ cDCs in a respiratory syncytial virus model (144). IL-25 promotes both the Th2 and the Th17 differentiation of T cells through activation of CD11c^+^ DCs (52, 145). So far, the impact of IL-33 on lung DC subsets has not been investigated.

### Plasmacytoid DCs

Plasmacytoid DCs sense viral infections through activation of TLR7 and TLR9, resulting in the production of large amounts of type I interferon (IFN-α), which is critical for a quick antiviral response. Thus, pDCs play a major role in respiratory viral infection and clearance (146, 147), in particular in the context of allergic asthma (148, 149). Of note, in contrast to the spleen, lung pDCs do not express TLR9 (150).

At steady state, pDCs are present at very low numbers in the lung, where they maintain tolerance to harmless inhaled antigens (92, 93). Upon allergen contact, the number of pDCs increases (151) partly due to the release of IL-15 by airway ECs (152). However, pDCs are poor allergen presenters in comparison to CD11b^+^ cDCs in different models of experimental allergic asthma (26, 92, 151).

Originally, pDCs were described as tolerogenic, as they can drive Treg cell differentiation in response to allergen uptake and migration to the lymph nodes (92, 93, 153). Specific depletion of pDCs, using a SiglecH-DTR system resulted in decreased tolerance (154). Interestingly, a specific subtype of pDCs, characterized by the expression of CD8α/CD8β, was the major inducer of Treg cells (39). CD8α^+^/CD8β^+^ pDCs express retinal dehydrogenases (RALDHs) resulting in the generation of retinoic acid, which is a metabolite critical for the development of Treg cells (39). In a recent study, the increased number of CD8α^+^/CD8β^+^ and CD8α^+^ pDCs has been associated with increased tolerance in a HDM-driven peptidoglycan recognition protein (Pglyrp1)-deficient mouse model (155). However, the dominant role of pDCs in the development of Treg cells has been challenged recently, since lung CD103^+^ cDCs but not pDCs showed increased expression of RALDHs after allergen administration (135).

In addition to the direct effect of pDCs on Treg cell development, they were also shown to act in *trans* by regulating the functions of mDCs (presumably a mixture of CD11b^+^ cDCs and moDCs) during the crosstalk with naive T cells (151, 156) in a mechanism that involves the regulation of B7 molecule expression (157). Administration of Flt3L increased the number of pDCs in the lung and promoted a strong anti-inflammatory response through an increase of the pDC/cDC ratio (158). This impact of pDCs in modulating cDC function has also been observed in pulmonary viral infections (159).

### Monocyte-Derived DCs

At steady state, the moDC subset is very difficult to discriminate from the CD11b^+^ cDC, due to the lack of a clear and universally used surface marker-based strategy. Markers to identify them encompass Ly6C (160), CD64, FcεR (26), and more recently, C5aR1 (42). Forty-eight hours after initial HDM exposure, CD11b^+^ CD64^+^ FcεR^+^ moDCs accumulate at high numbers in the lung. Seventy-two hours after HDM challenge, they peak in the draining lymph nodes (26). Although they are very efficient in antigen uptake, their potency to drive naive T cell proliferation at low allergen doses is lower than that of CD11b^+^ cDCs (26). More importantly, the production of cytokines and chemokines, such as CCL24 (eotaxin-2), CCL2, CCL4, CCL7, CCL9, and CCL12 that are critical for the activation and recruitment of eosinophils and monocytes in response to allergen challenge, has been suggested as the major function of moDCs (26).

It could be shown that the massive recruitment of moDCs to the lung is dependent on both the CCL2/CCR2 signaling axis and the formyl peptide receptor 2 (26, 161). Since the stimulation of a human bronchial EC line with the HDM associated protease Der p1 resulted in the release of CCL2, this mechanism was suggested to account for the recruitment of moDCs in HDM-driven asthma (162).

## Dendritic Cell Functions in Chronic Asthma

Many studies have focused on the role of pulmonary DCs in allergen sensitization and the acute phase of allergic inflammation. Much less is known about their role for the resolution of airway inflammation. Resolution encompasses various mechanisms, such as removal of apoptotic cells (efferocytosis), dampening of the inflammatory cytokine signals, increase of anti-inflammatory signals (including IL-10), production of protective lipid mediators (such as resolvins and protectins), and the expansion of Treg cells (163). Failure to perform these tasks together with a constant recall of the inflammatory Th2 response upon repeated allergen contact will lead to a chronic inflammatory state (164), resulting in remodeling of the airways. In the asthmatic lung, the resolution of the initial inflammatory insults is impaired. Although the mechanisms are poorly defined, they were proposed to include a decreased clearance of apoptotic cells (165) and a decreased secretion of resolvin E1 (166). Our knowledge about the contribution of the different DC subsets to the resolution of inflammation in the asthmatic lung is rather limited. CD103^+^ cDCs have been shown to remove apoptotic cells and to cross-present antigens in draining lymph nodes (41). CD103^−/−^ mice have a reduced capacity to resolve lung inflammation in allergic asthma models (136). Resolvin E1 inhibits the motility of skin DCs and reduces their capacity to drive T cell priming (167). In addition, BMDCs generated in presence of the resolvin E1 do not acquire chemokine receptor expression but trigger apoptosis of activated CD4^+^ T cells (168), suggesting that such DCs help clearing the excessive infiltration of Th2 cell in the inflamed lung.

The cellular aspects of airway remodeling include not only the recruitment of inflammatory cells, such as eosinophils, neutrophils, and mast cells, but also the uncontrolled expansion of fibroblasts and airway smooth muscle cells (169). DCs may actively contribute to airway remodeling. In an HDM-driven chronic asthma model, recurrent allergen contact triggers TSLP release from the airway epithelium, which leads to an increased surface expression of OX40L as well as CD80 and CD86 on CD11c^+^ DCs. In this model, neutralization of TSLP alleviated not only the Th2 inflammation but also the airway remodeling (161). Additionally, in an OVA-mediated model of chronic allergic asthma, DCs sustained Th2 activation and airway remodeling through the secretory phospholipase A2-V (sPLA2-V)-dependent eicosanoid generation in DCs (170). During the chronic phase of asthma, TGF-β is considered as a key regulator of remodeling (171). TGF-β secreted by fibroblasts has been shown to modulate the migration of DCs in the lung (172), which might be another way by which DCs control the remodeling of the airways. In conclusion, even though the presence and functions of DCs in chronic asthma have been underestimated so far, recent studies indicate that modulation of their function may play a critical role for the resolution of the inflammation and therefore may be critical for the remodeling of the airways.

## Lung Macrophage Subset Functions in Allergic Asthma

### The Dual Roles of AMs in the Regulation of Allergic Asthma

As outlined above, the term AM is frequently used to describe airway and AMs. In rats, the two populations have slightly different functions, in particular with regard to their enzymatic activities, phagocytotic potency, expression of surface markers CD163 and CD68 (21), and particle clearance (173). In mice, no data are available that show a difference in the modulation of allergic asthma by these two subsets.

Alveolar macrophages recognize and clear apoptotic cells and pathogens. In order to fulfill these tasks, they express a wide range of pattern-recognition receptors, the activation of which drives the strong production of reactive oxygen intermediates (174). Considering AMs only as garbage collectors that remove potentially dangerous self or non-self components from the alveolar space seems to be too simplistic. Instead, AMs are potent sentinels which sense inhaled pathogens that interact with each other and surrounding cells such as ECs. As already outlined above, AMs can be distinguished based on the strength of adherence to the epithelium. Strongly connected AMs express connexin 43. This expression not only allows a physical connection between AMs and ECs but also enables AMs to exchange information with surrounding AMs *via* a Ca^2+^ flux through connexin 43-containing gap junctional channels using ECs as the conducting pathway (127). On the one hand, this crosstalk suppresses secretion of proinflammatory chemokines, such as MIP-1a and CXCL1/5, by AMs and ECs; on the other hand, it leads to a diminished recruitment of proinflammatory neutrophils (127). These data are in contrast to the observation of Song et al., who reported AMs as the major source of IL-17 in an OVA model of experimental asthma (175). A possible explanation for this apparent conflict might be that Westphalen et al. studied the function of tightly adhered AMs (127), whereas Song et al. focused on total AMs (175). Moreover, immunosuppressive functions of AMs were also shown by Zaslona et al., who observed that depletion of AMs leads to a decrease of TGF-β production (112). Whether the underlying immunosuppressive mechanism is mediated by inducible Treg cells is still not entirely clear (2, 112, 176). This finding was supplemented by two reports, which showed an AM-mediated suppression of DC-driven antigen presentation and DC-mediated inflammation (115, 177). Despite these anti-inflammatory properties of AMs, most AMs present in the allergic asthmatic lung are predominantly M2-polarized macrophages (178), expressing M2 type-related proteins (arginase 1, YM1 and 2, resistin, and EAR-11), chemokines (CCL-8 and CCL-17), and metalloproteases (MMP-14 and ADAM-18) (179), thus acting mainly as proinflammatory cells. In order to combine both anti-inflammatory and pro-asthmatic properties of AMs, different models of activation and function of AMs have been described. One model suggested by Peters-Golden et al. proposes that AMs differentiate between direct and indirect uptake of pathogenic particles, hence leading to tolerance or inflammation (180). While the indirect uptake of pathogens by phagocytosis of infected apoptotic cells induces anti-inflammatory functions, the direct uptake of opsonized microbes induces inflammation (180). Hussell et al. noted that the exact location of AMs in the alveoli or the mucous layer of bigger airspaces and the microenvironment, which is largely shaped by the microbial flora, needs to be taken into account in order to fully appreciate AM functions in the pulmonary tissue (22).

### The Enigmatic Roles of IMs in the Regulation of Allergic Asthma

In contrast to AMs, less is known about the functions of IMs. After migration into the lung, the majority of circulating monocytes differentiates into IMs and starts expressing COX-2 and MHC-II (34). Depletion of circulating monocytes resulted in a decreased asthmatic phenotype, suggesting a proinflammatory function of IMs during asthma development, which is in part mediated by IL-5 (112). Moreover, IMs were shown to express IL-17 but were not increased in allergic asthma as compared to AMs (175). They also express CCL-11 but not CCL-24 (179). Depletion of AMs by clodronate in IL-13-driven airway inflammation resulted in an increased number of IMs in the tissue (181). Also, tissue-resident macrophages have been shown to induce Treg cells in response to uptake of harmless antigens (33) and to produce IL-10 following airway challenge with low doses of LPS (31). However, not all monocytes that enter the lung differentiate into IMs. Instead, they can keep their monocytic phenotype, take up antigens, and migrate *via* CD62L to draining lymph nodes, suggesting that monocytes can mimic IM functions *in vivo* (34). However, it should be noted that different approaches were used in these studies to define IMs. It is, therefore, not clear if the same cell population was examined.

## Conclusion

Taken together, the functions of the different DC subsets during the onset of allergic asthma are very complex and highly dependent on both the nature and the levels of inhaled allergen. Moreover, the composition of the local microenvironment, defined by the surrounding cells and consequently by DC localization within the lung, also influences DC activation and T cell differentiation. During the past decade, evidence has accumulated that within the group of pulmonary DC subsets, CD11b^+^ cDCs mainly drive the development of maladaptive Th2/Th17 immune responses, whereas moDCs are recruited to orchestrate the local inflammation in the lung. Since CD103^+^ cDCs can sample the airway lumen without epithelial damage, they may serve as an important interface for signal transmission to neighboring cells, thereby licensing such cells to exert inflammatory functions. However, their role in Th2 skewing is still controversial. Furthermore, several studies have demonstrated a role for CD103^+^ cDCs and pDCs in keeping pulmonary tolerance. In addition, there is increasing evidence that the different DC subsets also contribute to the resolution of inflammation in allergic asthma. At this point, a holistic view of the individual roles of each DC subset is still missing, but we are beginning to assemble the required information (see Figure 3). Finally, recent studies suggest that pulmonary macrophages have been illegitimately neglected in asthma. In fact, they seem to regulate the development of maladaptive T cell immunity, maintenance, and/or resolution of asthmatic inflammation. Interestingly, their localization in the alveolar space or their attachment to ECs seems to determine their pro- or anti-inflammatory functions. The role of IMs in this scenario is ill-defined. Clearly, more studies are needed that define their spatiotemporal distribution and the cells with whom they interact in the lung during the different phases of allergic asthma. In light of the emerging contributions of AMs to the asthmatic phenotype, we would not be surprised to also see critical roles of IMs in asthma development.

## Author Contributions

Writing of the text: FH, FE, and IS; drafting of the manuscript: PK, IL, JK, and YL; design of figures: FH, FE, IS, JK, and YL; revision: FH, FE, IS, PK, IL, JK, and YL; final approval: FH, FE, IS, PK, IL, JK, and YL; and agreement to be accountable: FH, FE, IS, PK, IL, JK, and YL.

## Conflict of Interest Statement

The authors declare that the research was conducted in the absence of any commercial or financial relationships that could be construed as a potential conflict of interest.
